# Nanomicelles: Types, properties and applications in drug delivery

**DOI:** 10.1049/nbt2.12018

**Published:** 2021-02-02

**Authors:** Anamika Bose, Debanwita Roy Burman, Bismayan Sikdar, Prasun Patra

**Affiliations:** ^1^ Amity Institute of Biotechnology Amity University Kolkata West Bengal India

## Abstract

Nanomicelles are self‐assembling nanosized (usually with particle size within a range of 10 to 100 nm) colloidal dispersions with a hydrophobic core and hydrophilic shell. Owing to its size, solubility, customised surface or its exposure to the environment, nanomicelles show some unique or novel characteristics, which makes it multifunctional and thus makes its use indispensable in biomedical application and various other fields. This review presents the unique properties of nanomicelles that makes it different from other particles and paves its way to be used as drug delivery agent and many other biological uses or applications. It also emphasises on the drug encapsulation ability of the nanomicelles and different technique of drug loading and delivery along with its advantages and disadvantages.

## 1 INTRODUCTION

Micelles are amphiphilic colloidal structures having a particle diameter ranging from 5 to 100 nm. Micelles constitute of molecules having two regions of different affinities for water. The amphiphilic molecules forming micelles associate at certain temperatures and certain concentrations. The critical micelle concentration is the concentration at which aggregation begins and the micelles are formed. Similarly, the critical micellar temperature is the temperature at which the micellar molecules aggregate and below which no micelles are formed and exist as monomers. The number of monomer molecules forming a micelle is known as the aggregation number of the micelle. The aggregation of the amphiphilic molecules leading to the formation of micelles occurs due to the removal of the hydrophobic fragments of the micelles from the aqueous environment and the formation of hydrogen bonds in water which leads to a decrease in free energy of the system. Micelles are used as drug carriers in various pharmaceutical industries as they can carry lipophilic drugs within its core while the micellar surface binds polar molecules. Polymeric micelles are used in therapeutics as they provide an improved solubility and hence better intestinal permeability of micelles. Polymeric micelles are formed of amphiphilic block copolymers [1]. Polymeric micelles have long circulating characteristics and significant tumour accumulation which provide an important use in tumour targeting [2]. In contrast with the surfactant micelles, polymeric micelles are usually more stable, have a lower critical micelle concentration (CMC), a slower dissociation rate and achieving high drug accumulation at the target. The narrow size range of the micelles are similar to that of viruses and lipoproteins which is a crucial and significant factor in determining their body arrangement when an enhanced permeation retention effect (EPR) is involved [3].

High drug toxicity acts as a barrier to proper treatment as it leads to side effects. An example can be given of the cancer drugs, where these drugs act on both cancerous as well as the healthy tissues. Side effects range from nausea, hair‐loss, neuropathy, neutropenia as well as kidney failure [4]. The drug–nanocarrier complex thereby is seen to be highly selective and specific increases the amount of drug delivered to the target affected tissue and decreases the amount at the unwanted sites [5]. Many liposomal formulations for anticancer drugs have been already approved. For example, the drug Doxil is a liposomal formulation of the drug anthracycline drug doxorubicin which is used as an anti‐cancer drug in AIDS‐related Kaposi sarcoma and multiple myeloma [6]. This drug is more advantageous than the free drug doxorubicin as it offers more efficacy and low cardiotoxicity [7].

In addition, nanomicellar drugs are observed to overcome drug resistance by the increase of drug accessibility and drug sensitivity through high local drug concentration achieved at tumour sites through the enhanced permeation and retention (EPR) effect [8]. Among all other systems for nanotechnology based drug delivery systems such as liposomes, nanotubes and nanomicelles [3], nanomicelles are more advantageous for cancer therapy, such as high drug loading capacity for effective therapeutic potency and small size for deep tumour tissue penetration [9].

Nanomicelles are used in the process of targeted drug delivery which allows a greater depth of tissue penetration and increases the bioavailability of the drugs [10]. Regular micelles orient themselves with an interior hydrophobic core and an exterior hydrophilic part when placed in a polar solvent. They are used for the encapsulation of poorly soluble drugs and hence they help in the solubility and hence the bioavailability of the drugs [11]. The reverse micelles on the other hand are oriented in an opposite fashion with the hydrophobic regions facing towards the exterior and the hydrophilic regions towards the interior when placed in a non‐polar solvent. They are used for the encapsulation and delivery of hydrophilic drugs and proteins such as lysozymes [12] and solutes such as trypan blue [13] and fluorescein [14]. Sustained drug release is required for the treatment of chronic diseases, infectious diseases and also cancer which is of a greater concern today. Few of the approaches for sustained drug release from the micelles include synthesis of prodrug, usage of novel polymers, layer by layer micellar assembly on solid support, reverse micelle formation, drug polymer conjugate micelles preparation and also development of polymer films which form micelles in vivo. The small size of the micelles, provide a greater advantage for drug targeting than the larger systems [15]. The aggregates formed from the amphiphilic molecules provide many advantages like increasing the solubility of sparingly soluble compound, stability, ease of sterilisation. The lipophilic molecules are arranged inside the hydrophobic core of micelles formed by the van der Waals forces [16], whereas the outer hydrophilic layer protects from steric hindrance and the construct from being recognised and captured by the Reticular Endothelial System (RES) hence providing a longer circulation time [17], helps in driving to conjugation with a ligand for active targeting [18].

## 2 PREPARATION OF NANOMICELLES

Nanomicelles are formed by the self‐assembly of amphiphilic molecules in aqueous media. Amphiphilic molecules are molecules containing both a polar or hydrophilic and non‐polar or hydrophobic part. These particles are formed by the orientation of the hydrophobic part away from the solvent and forming the interior and the hydrophilic polar part as the exterior and orienting itself towards the solvent, when placed in a hydrophilic or polar solvent. The hydrophobic part is in the interior as it is unable to interact with the aqueous solvent whereas in the contrary, hydrophilic part or the polar part is able to interact with the solvent. The aggregate of the amphiphilic molecule in this orientation is known as regular micelles. When placed in a non‐polar or hydrophobic solvent, the amphiphilic molecules arrange themselves in an opposite orientation where the hydrophobic part is in the exterior and the hydrophilic portion is in the interior. These kinds of micelles are known as reverse micelles [19].

Nanomicelles are prepared from surface active agents such as surfactants and synthetic block copolymers. Amphiphilic monomers exist either in ionic, non‐ionic or zwitterionic forms. Ionic surfactants may carry either a positive (cation) or a negative (anion) charge. Anionic surfactants include sodium dodecyl and cationic surfactants include dodecyl trimethyl ammonium bromide [20]. Non‐ionic surfactants are neutral such as n‐dodecyl tetra (ethylene oxide) whereas zwitterionic surfactants carry both negative and positive charges such as dioctanoyl phosphatidyl choline. Polymeric micelles can be arranged as linear diblock (A‐B), triblock (A‐B‐A), pentablock (A‐A‐B‐A‐A), and branched types. In the arrangements of block copolymers, A and B represent any of the polymers, but are not limited to poly(lactic acid), poly(ethylene glycol) (PEG), poly(L‐lysine) (PLL), polyethylene oxide, poly(D,L‐lactic acid), polypropylene oxide, polyglycolic acid, poly(aspartic acid), poly(glutamic acid), poly(L‐lysine), and poly‐(histidine) [21].

## 3 UNIQUE PROPERTIES OF NANOMICELLES

### 3.1 Structural stability

Nanomicelles possess both Thermodynamic and Kinetic stability which is due to the entanglement of polymer chains present in the inner core of the micelle. Thermodynamic stability is attained by a nanomicelle when its copolymer concentration exceeds its CMC (Critical Micelle Concentration), which is influenced by the hydrophilic‐lipophilic balance (HLB) of the copolymer [22]. Generally, nanomicelles show very low CMC values which ranges from 10^−6^ to 10^−7^ M, which are smaller than that of micelles formed from low‐molecular weight surfactants, which enables it to retain its micellar structure upon series of dilution [23]. Kinetic stability becomes more important factor when the conditions for drug delivery are not at equilibrium. And moreover, Kinetic stability comes into action when copolymeric concentration falls below its CMC. Kinetic energy for micelles is high for the core structure. Thus, nanomicelles disassemble much slowly because of their kinetic stability when the concentration is less than that of CMC. This slow dissociation due to its structural stability allows the nanomicelles to retain the integrity and maintain its drug content till the time it reaches its target site, which enhances bioavailability of the drug [24].

### 3.2 Ability to solubilise hydrophobic drug

Nanomicelles are considered the best pharmaceutical carriers which solubilise hydrophobic drugs. Hydrophobicity, is one of the major limiting factors which can formulate clear aqueous solution with concentration of the drug which is sufficient for attaining therapeutic levels. Nanomicelles have the capability to solubilise hydrophobic drugs by entrapping the drugs within a mixed micellar hydrophobic core, while the shell is composed of hydrophilic chains extending outwards which results in a clear aqueous solution [25].

### 3.3 Physicochemical properties

Nanomicelles are amphiphilic copolymers which are usually block copolymers [26]. Block copolymers can be diblock or triblock copolymers. Generally, diblock copolymers are mainly A‐B type, where A represents Hydrophilic part and B represents hydrophobic part. Whereas triblock polymers are of two types – ABA type and ABC type. AB and ABA type is widely used to make nanomicelles [27, 28]. These arrangements enhance drug solubility and increases loading efficiency. The inner core made of hydrophobic blocks are stabilised by hydrophobic interaction, or by electrostatic interaction which results from the macromolecules of opposite charges, which form PIC or Polyion Complex micelles. Apart from these, it is also formed by metal‐ligand binding and also by hydrogen ion bonding [29, 30, 31]. The outer shell which is hydrophilic in nature has steric stability and plays an important role in in vivo condition due to interaction with the cells [32]. Thus; micelles can self‐assemble in water, enhances drug solubility, and remains stable in the gastro‐intestinal tract because of its structure.

### 3.4 pH sensitivity

Drug release depends upon intracellular signals where pH‐responsive systems are of great importance. Micelles, polybases, and polyacids are the building blocks that impart pH sensitivity to the release of drug [33]. Amine, the basic core is uncharged and thus renders to be hydrophobic at high pH and becomes hydrophilic upon protonation at low pH, whereas, carboxylic acid, the acidic core is uncharged at low pH and upon being protonated becomes negatively charged at high pH. Thus, protonation can be responsible for destabilising micelles. pH must be above pKa of the protonable group for the formation of the nanomicelle. But, as soon as the pH falls below the pKa value, hydrophilicity and electrostatic repulsion are increased because of the ionisation of the polymers, which leads to the destabilisation of the micelle which in turn leads to controlled drug release [34]. Thus, absorption of drug in the intestine can be promoted by making use of this pH gradient [35].

### 3.5 Mucoadhesive property

Generally, nanoparticles involved in oral medication adheres to mucous and crosses the mucosal layer, where the particles disintegrate thus leaving behind the drug bound to the mucosal layer which is cleared by mucus clearance mechanism which leads to low bioavailability of the drug [36]. By this property, mucosal retention increases the transit time in the Gastro‐Intestinal tract, which enhances the time for drug release. Moreover, mucoadhesive nanomicellar polymers swell and fill the cervices of the mucous membrane, which increases the effective surface area which leads to the high local concentration of drugs [37]. Lastly, bioadhesion increases the drug concentration gradient for contact of particles with mucosal surface [38]. Studies reveal that, intestinal mucosa, which is negatively charged due to glycocalyx, attracts polymicelles which are positively charged. Thus, particle mobility depends upon surface charges. It was demonstrated by Crater and Carrier anionic particles diffuse 20‐30 times faster than that of cationic particles [39].

### 3.6 Specific binding ability

Targeted drug delivery is achieved by enhanced Permeability and retention (EPR) effect, by making micelles of stimuli‐responsive amphiphilic block‐copolymers, or by attaching specific targeting ligand molecules to the micelle surface. Immuno‐micelles, which can be prepared by coupling monoclonal antibody molecules to *p*‐nitrophenyl carbonyl groups on the water‐exposed termini of the micelle corona‐forming blocks, demonstrate high binding specificity and target ability [40].

### 3.7 Light sensitivity

Studies indicate that hydrophilicity and hydrophobicity can be altered on exposure to light. Thus, light can trigger disintegration of nanomicelles and thus release of drug [18, 41].

## 4 TYPES OF MICELLES

### 4.1 Regular micelles

Regular micelles are self‐assembled structures of amphiphilic copolymers in aqueous medium. They are prepared in an aqueous solution and have a hydrophilic region on the outside or the aqueous environment and a hydrophobic region on the inside. For example, PEG‐polylactic acid, PEG‐PLGA, polyethylene oxide‐poly(propylene oxide).

### 4.2 Reverse micelles

They are the self‐assembled structures of amphiphilic copolymers in non‐aqueous medium. They are prepared in an organic medium and have a hydrophobic region on the outside and a hydrophilic region on the inside. For example, Phosphazene micelles in chloroform, PCL‐P2VP micelles in oleic acid.

### 4.3 Unimolecular micelles

**FIGURE 1:**
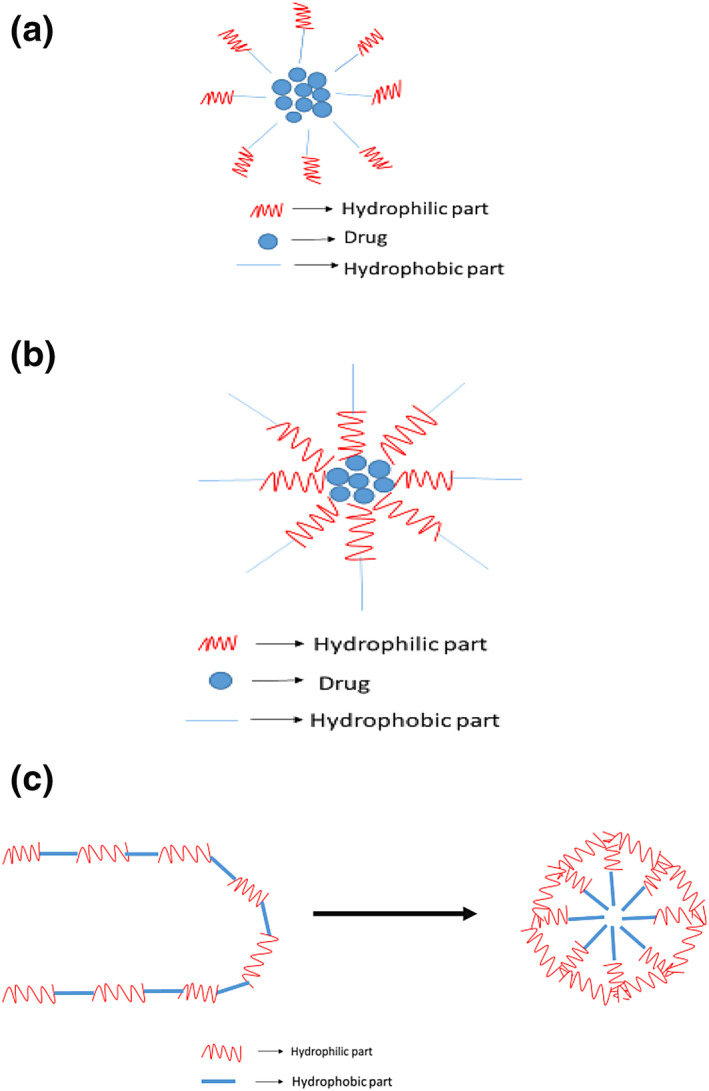
Regular micelle; (b) reverse micelle; and (c) unimolecular micelle

These polymers have many hydrophilic and hydrophobic regions in one molecule which enables in the self‐assembly of one molecule into a micelle. These are formed from amphipathic molecules for example, Core (Laur) PEG micelles in aqueous medium. They possess unique single molecular architectures that can maintain the stability when subjected to extreme environmental changes like high dilution and changes in temperature, pH, ionic strength, and so forth [42] (Figure 1).

## 5 METHODS OF DRUG DELIVERY USING NANOMICELLES

Nanomicelles are colloidal structures (5–100 nm) which are formed from the amphiphilic monomers having two parts, a small hydrophobic head and a long hydrophilic tail. Since it has small size, it is considered to me more ideal for drug delivery to take place, and also its Nano structure provides its structural stability. Drugs can be encapsulated inside a micelle and the micelle would act as a carrier to deliver the drugs to the targeted tissue. As mentioned earlier, since it is Nano scale, it can pass through any capillary or vascular system and it reaches the target very quickly. The drug release is mainly affected by the extent of micelle moieties which results in slower release and high precision. Some good examples of micelles used to carry chemotherapy are Doxorubicin and Paclitaxel which are now approved for clinical trial [43].

The methods that are widely used to load drugs inside micelles are discussed in the following sections.

### 5.1 Oil‐in‐water (O/W) emulsion techniques

Nano emulsions are a non‐equilibrium, heterogeneous system consisting of two immiscible liquids in which one liquid is dispersed as droplets in another liquid. Emulsions with nanoscopic droplet sizes (typically in the range of 20–200 nm) are often referred to as submicron emulsions. In a Nano emulsion, the oil droplets serve as the reservoir for hydrophobic drugs**.** The most widely used oil molecules include saturated and unsaturated fatty acids, fatty acid esters, and soybean oils. Surfactant molecules play a key part in stabilising the Nano emulsions. Non‐ionic or amphoteric surfactants such as poloxamer, lecithin, and Tween 80 are commonly used. Combinations of various surfactants have also been used to control droplet size and improve the stability of Nano emulsions [44].

### 5.2 Water‐in‐oil‐in‐water (W/O/W) emulsion techniques

Freshly prepared W/O/W emulsions are analysed using an optical microscope at room temperature. Samples were placed on microscopic slides and then carefully covered with a cover slip to minimise destruction of the emulsion structures. The microstructures of the W/O/W emulsions were observed using an oil immersion objective (100× magnification), and an appropriate light intensity was selected to reduce sample heating [45].

### 5.3 Direct dialysis

The dialysis method involves addition of small amounts of water to the solution of polymer and drug in a water‐miscible organic solvent like dimethyl formamide with stirring followed by dialysis against an excess of water for several hours using a dialysis bag for the removal of organic solvent [46, 47].

### 5.4 Co‐solvent evaporation

According to modern study, this type of evaporation method is carried out by dissolving appropriate amount of any drug like Simvastatin (SIM), in 100 ml of methanol and appropriate amount of hydroxypropyl methylcellulose [HPMC (K3LV)] in appropriate volume of distilled water and mixed both solutions, which produces clear solution. The clear solution evaporated at specific pressure and specific temperature for half an hour in rotary evaporator.

### 5.5 Spray drying

Generally, the solvent evaporation is carried out by using spray dryer. The solutions are prepared by dissolving appropriate amount of drug in appropriate volume of methanol and specific amount of surfactant in specific volume of distilled water and mixing both solutions, which produces a clear solution. The solvent is evaporated at inlet 110°C and outlet 60°C, feed pump speed 10 ml per minute and aspiration 45%. The spray dried mixture of drug with is obtained in 20–30 min.

Among all these above mentioned drug encapsulation methods, O/W, direct dialysis and co‐solvent evaporation are well compatible for encapsulating hydrophobic drugs where as in case of W/O/W is often preferred for the encapsulation of more hydrophilic compounds [48].The drug will not release until all the Nano micelles get accumulated in the targeted site since the micelles are stable enough in their Nano scale and also if intravenous administration is taken place.

## 6 NANOMICELLE–MEDIATED DRUG LOADING AND RELEASE

In general, there are three major methods for loading drugs into polymer micelle cores; they are discussed in the following sections [49].

### 6.1 Chemical conjugation

In this kind of technique, a drug is chemically conjugated to the core resulting to form a block of co‐polymer via a carefully designed pH or enzyme sensitive linker that can be cleaved to release a drug in its active form within a cell. The conjugate then acts as polymer prodrug which self‐assembles into a core‐shell structure. The nature of the polymer‐drug linkage and the stability totally depends on the rate of drug release. The nature of the polymer‐drug linkage and the stability of the drug conjugate linkage can be controlled to influence the rate of drug release, and therefore, the effectiveness of the prodrug. For instance, according to a recent work, pH‐sensitive polymer micelles of PEO‐b‐poly(aspartate hydrazone doxorubicin), in which doxorubicin, was conjugated to the hydrophobic segments through acid‐sensitive hydrazone linkers that are stable at extracellular pH 7.4 but degrade and release the free drug at acidic pH 5.0 to 6.0 in endosomes and lysosomes [2]. Doxorubicin was conjugated to the poly (aspartic acid) chain of PEO‐b‐poly (aspartic acid) block copolymer though an amide bond. Adjusting both the composition of the block copolymer and concentration of conjugated doxorubicin led to improved efficacy, as evidenced by a complete elimination of solid tumours implanted in mice. It was later determined that doxorubicin physically encapsulated within the micellar core was responsible for antitumour activity. This finding led to the use of PEO‐b‐poly (aspartate doxorubicin) conjugates as Nano containers for physically entrapped doxorubicin [2].

### 6.2 Physical entrapment

The physical incorporation or solubilisation of drugs within block copolymer micelles is generally preferred over micelle‐forming polymer‐drug conjugates, especially for hydrophobic drug molecules. Indeed, many polymers and drug molecules do not contain reactive functional groups for chemical conjugation, and therefore, specific block copolymers have to be designed for a given type of drug. In contrast, a variety of drugs can be physically incorporated into the core of the micelles by engineering the structure of the core‐forming segment. In addition, molecular characteristics (molecular weight, composition, presence of functional groups for active targeting) within a homologous copolymer series can be designed to optimise the performance of a drug for a given drug delivery situation. This concept was introduced by our group in the late 1980s and was initially called a ‘micellar micro container’, but is now is widely known as a ‘micellar nanocontainer’. Haloperidol was encapsulated in Pluronic block copolymer micelles, the micelles were targeted to the brain using brain‐specific antibodies or insulin, and enhancement of neuroleptic activity by the solubilised drug was observed.

### 6.3 Polyionic complexation

Charged helpful operators can be consolidated into square copolymer micelles through electrostatic associations with an oppositely charged ionic portion of square copolymer. Since being proposed autonomously by Kabanov and Kataoka in 1995, this methodology is presently broadly used for the joining of different polynucleic acids into square ionomer edifices for creating non‐viral quality conveyance frameworks. Ionic square lengths, charge thickness, and ionic quality of the arrangement influence the development of stable square ionomer edifices and, in this way, control the measure of the medication that can be joined inside the micelles. The pH‐ and salt‐affectability of such square ionomer micelles give a one of a kind chance to control the activated arrival of the dynamic restorative agent. Furthermore, square ionomer edifices can take an interest in the polyion exchange responses which are accepted to represent the arrival of the helpful specialist and DNA in a functioning structure inside cells. Several exhaustive surveys can be found in the writing that emphasis on square ionomer micelles as medication and quality conveyance frameworks. Likewise physicochemical parts of the DNA buildings with cationic square copolymers have been additionally as of late evaluated.

## 7 ADVANTAGES OF NANOMICELLES IN DRUG DELIVERY SYSTEM

Nanomicelle‐based drug delivery system has some advantages over the classical drug delivery system mainly because of its size and structural composition. Some of the advantages are as follows:

- Nanomicelles being an amphiphilic molecule, the hydrophobic drugs bind to the hydrophobic core of the nanomicelle and thus, this result into production of clear aqueous solution, increasing the solubility of lipophilic drug by several folds. [21, 49, 50, 51, 52]. For example, polymeric nanomicelle of Efavirenz, an antiretroviral has a solubility of 34 mg/ml, which otherwise normal Efavirenz (non‐nanomicelle) being poorly soluble in water would have had solubility of only about 4 µg/ml. Thus, nanomicelles entrap hydrophobic drugs and increase its solubility [51].
- Nanmomicelles are target specific due to their active targeting capability, which leads to maximum delivery and minimum side effects. Specific targeting is due to their conjugation with target moieties. The interactions can be with specific surface receptor, transporter protein, applied signal protein or the phage fusion proteins [52]. Ahn et al. prepared a fragment of antibody which was conjugated with nanomicelles loaded with platinum which targets pancreatic cancer on tumorxenografts [53, 54].
- Nanomicelles release drugs on being triggered by certain stimulus such as pH, temperature, which leads to higher selectivity and lower toxicity. Drug remains entrapped inside nanomicelles under normal physiological condition (pH 7.2), but under acidic condition, the drugs or the therapeutic agents are released in the targeted part of the body as tumour cells, lysosomes and endosomes are acidic in nature. This pH gradient acts as the stimulus which leads to the targeted release of the drug in the desired portion [55, 56, 57]. For example, Bae et al. prepared pH sensitive Polyethylene Glycol(PEG)‐Poly Aspartate Hydrazone Adriamycin‐loaded nanomicelles, which releases at low pH due to hydrazine linker which is acid sensitive [58].
- Nanomicelles are made of polymeric nanoparticles which are hydrophobic as well as biodegradable. These biodegradable nanoparticles act as the local depot of drugs which are accumulated at the targeted site. This depot acts as the source of continuous supply of therapeutic agent or drug encapsulated in the nanomicelle at the diseased site, for example, solid tumours [59].
- Nanomicelles (>1 µm) because of their size are better suited for intravenous drug delivery. 5–6 µm is the size of the diameter of the smallest capillary in the body. Thus, nanomicelles which are smaller than the smallest capillary of the body ensure proper delivery of the drug into the bloodstream without the occurrence of embolism [59].

## 8 NANOMICELLE‐MEDIATED DRUG DELIVERY FOR SEVERAL THERAPEUTIC TREATMENT

### 8.1 In cancer treatment

**FIGURE 2:**
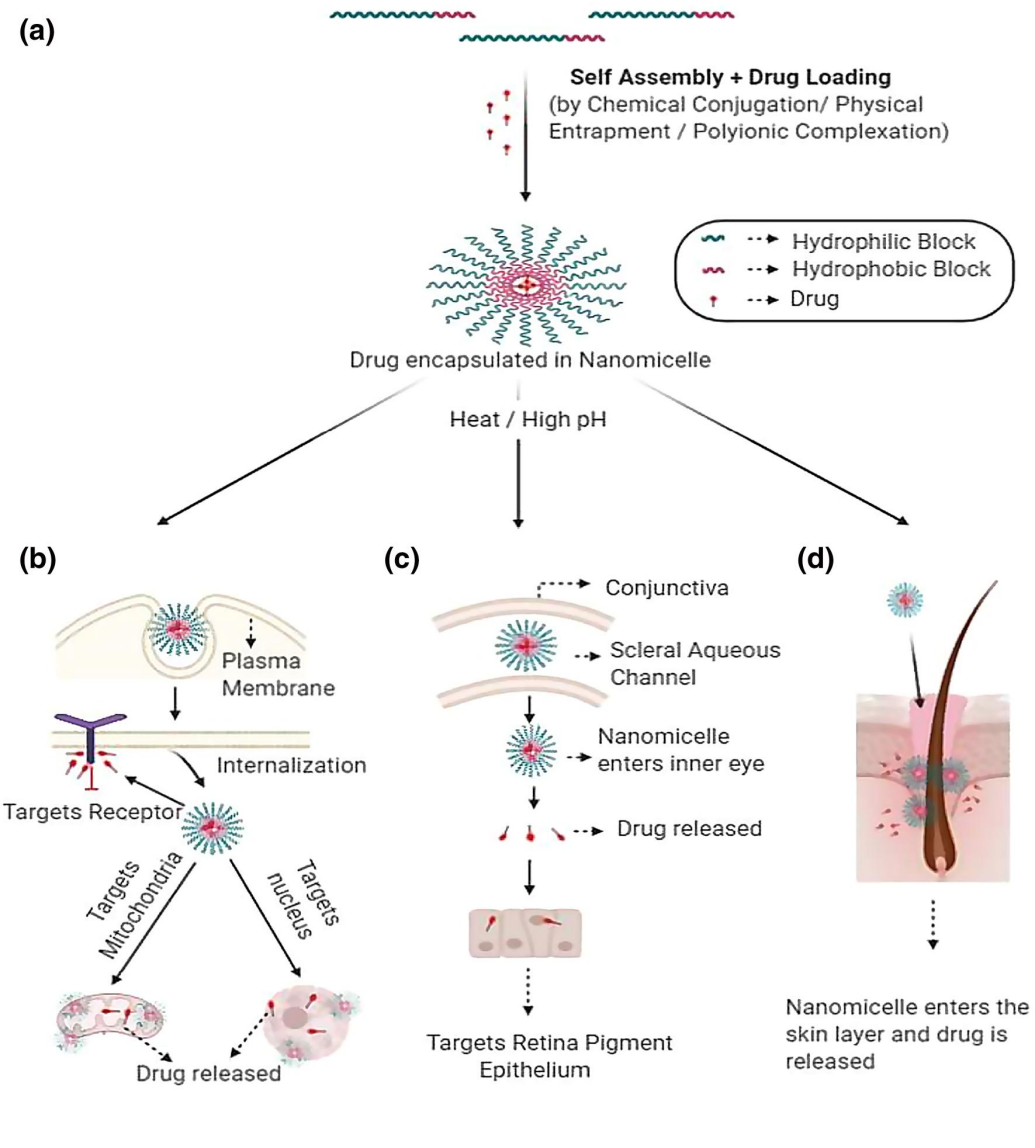
Schematic representation of (a) The formation of nanomicelle by the self‐assembly of amphiphilic molecule, with subsequent loading of drugs, thereby forming drug encapsulated nanomicelle; (b) The entry of the nanomicelle with subsequent release of drugs, targeting the mitochondria, nucleus and cell membrane bound receptor, thereby acting as potential therapeutic in treating cancer; (c) The entry of the nanomicelle in the inner part of the eye and releasing the loaded drug in the retinal pigment epithelium crossing the scleral aqueous channel thereby being highly efficient for the treatment of occular diseases; (d) Nanomicelle penetrating the hair follicles and releasing the drugs in the skin lipids, bypassing the skin barrier to treat skin diseases

Owing to several physico and biochemical advantages over other nanocarriers, polymeric micelles self‐assembled from amphiphilic block copolymers have led to great interests for targeted cancer drug delivery. They have a size of 20–100 nm, a hydrophobic core for efficient drug loading for poorly water‐soluble drugs and a hydrophilic shell to provide colloidal stability and intrinsic sheath effect [59, 60]. Biodegradable polymeric micelles are moreover ideal for targeted and controlled drug delivery of hydrophobic anti‐cancer drugs which include paclitaxel (PTX) and doxorubicin (DOX) [23, 61]. These nanocarriers (i) enhance water solubility of the anti‐cancer drugs; (ii) can prolong drug circulation time; (iii) are able to passively target tumour tissues via the EPR effect [62]; **(**iv) improve bioavailability; and (v) possess great biocompatibility and are also degradable into non‐toxic products in vivo which can be absorbed and excreted further from the human body. Micelles are a valuable means to control drug release and also prevent long term toxicity due to the drug's accumulation in the human tissues, due to its nature of biodegradability. Several anti‐cancer drugs have been approved in places like Japan, South Korea, UK, and USA [63] for example, Genexol‐PM, a PTX formulation based on biodegradable poly(ethylene glycol)‐b–poly (D,L‐lactide) (PEG‐PLA) copolymer micelles is approved for the treatment of breast, ovarian, and lung cancers [64]. The conventional methods used for cancer therapy include surgical resection of the tumour, chemotherapy and radiotherapy. Chemotherapeutic agents lead to non‐specific targeting of both cancer and healthy cells due to their cytotoxicity and non‐specificity. Hence, they lead to severe side effects. Polymeric micelles have proved to be an efficient drug carrier which helps in indiscriminate biodistribution to both normal and tumour tissues due to their low molecular weight [65]. Moreover, most of the anticancer drugs are water insoluble which results in poor absorption and low bioavailability hence a carrier is required which carriers the drug to the tumour (Figure 2).

### 8.2 In eye treatment

There has been a great development of nanomicellar formulation based technology for ocular drug delivery. The advantages include their high drug encapsulation capability, ease of preparation, small size and hydrophilic nanomicellar corona generating aqueous solution. Micellar formulations enhance the bioavailability of the drugs in the ocular tissues [66]. For example, dexamethasone‐loaded nanomicelles were developed by employing copolymers of poly hydroxyl ethyl aspartamide [PHEAC (16)] and PEGylated PHEAC (16) for the delivery of the anterior segment of the eye [67]. Researchers are studying to formulate a drug for diseases posterior segment of the eye. This method of drug delivery appears to be a potential pharmaceutical carrier for the topical administration of hydrophobic drugs. This technology is highly patient compliant and highly efficient for the treatment of age related ocular diseases such as macular degeneration, diabetic retinopathy, diabetic macular oedema, and posterior uveitis [68].

### 8.3 In skin treatment

The skin barrier inhibits the drugs from penetrating easily, as the skin acts highly refractive to hydrophobic and hydrophilic compounds. The new strategy for skin penetration is by the use of nano‐sized carriers for drug delivery [69]. Nanocarriers encapsulate pharmaceutical ingredients to perform features such as penetrate the hair follicles, interacting with skin's lipid to transport. There is high permeation through all routes including intracellular, intercellular and the hair follicle shafts in *trans*‐appendage pathway due to their high surface to volume ratio [70]. Several non‐toxic and biodegradable synthetic or semi‐synthetic polymers such as poly lactic acid (PLA), poly (lactic‐co‐ glycolic acid) (PLGA), poly (e‐caprolactone), chitosan have shown great results in topical drug delivery. The polymeric Nano carriers have shown merits in controlled release through modification of the polymer composition and reducing irritation due to direct contact of drug with skin [71, 72, 73].

## 9 CONCLUSION

Self‐assemblies of amphiphilic block copolymers have many interesting features from both fundamental and applied viewpoints. In particular, polymeric micelles formed from amphiphilic block copolymers in aqueous, have been extensively investigated as typical self‐assemblies. We focussed on polymeric micelles with amphiphilic block copolymers in aqueous media by employing systematic method. The basic principles for the formation of polymeric micelles through the organisation of block copolymers in aqueous and the physicochemical properties of drug‐loaded polymeric micelles have been briefly described. In addition, biological application of polymeric micelles has been given in last section. Nanomicelles have proven its worth and contribution in the Biomedical field as well. The field of nanomicelles has also brought up schemes for detecting cancer and diagnosing it. Poly‐b‐cyclodextrin inclusion‐induced formation of two‐photon fluorescent nanomicelles for biomedical imaging has also been seen to contribute the scientific society in the field of Biomedical field. It showed not only high stability but also high photostability and high cell‐permeability. The same technique is also used in detecting cancer cells and tissues. Nanomicelles have also been used to deliver plasmid DNA by developing polyplex structure in accordance with PEG. The polyplex nanomicelles systems have the advantage of high flexibility in terms of molecular design for specific applications. Once their safety and efficacy for gene delivery are established, a number of clinical applications can be envisioned using nanomicelles.

Without any doubt, the primary reason for using polymeric systems is the ease and simplicity with which they can form ordered nanoscale structures in aqueous via self‐assembly, meaning that expected shapes and sizes can be obtained without an additional trigger. In conclusion, we believe that control of the property of such polymeric nanomicelles as drug carrier is an addressable challenge even though the use of these polymeric micelles is still in its infancy for practical clinical applications. Therefore, this research field provides opportunities for chemists, physicists, biochemists, and so forth to develop systems that may eventually match in sophistication and precision biological structures elaborated by nature.
